# Effects of green oak acorn (*Quercus ilex*) intake on nutrient digestibility, lamb growth, and carcass and non-carcass characteristics

**DOI:** 10.5194/aab-65-113-2022

**Published:** 2022-03-11

**Authors:** Ilyes Mekki, Samir Smeti, Hadhami Hajji, Mokhtar Mahouachi, Naziha Atti

**Affiliations:** 1 Animal and Forage Productions Laboratory, University of Carthage, National Institute of Agronomic Research of Tunisia, rue Hédi Karray 2049, Ariana, Tunisia; 2 Livestock and Wildlife Laboratory, Arid Regions Institute (IRA), University of Gabes, 4119 Médenine, Tunisia; 3 Laboratoire Appui à la Durabilité des Systèmes de Production Agricole dans la Région du Nord-Ouest, ESAK, Le Kef, Tunisia, University of Jendouba, 7100 Jendouba, Tunisia

## Abstract

The green oak (*Quercus ilex*) plays an important role in forest ecology when oaks are the dominant species or are plentiful. The use of acorns as an alternative to barley for livestock feeding can be beneficial for breeders. The aim of this study was the evaluation of the acorn intake by lambs in two stages, suckling and fattening, on growth, diet digestibility, carcass and non-carcass characteristics. For this, 32 lambs were used. During the suckling period, 16 lambs were reared on range pasture, supplied by barley (S-Ba), the other 16 on forest pasture and supplied by acorns (S-Ac). During the fattening period, lambs were assigned to concentrate based either on barley (F-Ba) or acorn (F-Ac) resulting in eight animals per suckling treatment per fattening treatment. The feed intake, diet digestibility and lamb growth were recorded. At 90 d of fattening, all animals were slaughtered and carcass traits studied.

The main results show that the incorporation of acorn in concentrate was without effect on digestibility of organic matter, crud protein and neutral detergent fibre. The nitrogen balance was positive for animals fed barley concentrate or acorn one ( >8 g d ${}^{-1}$ ). The lamb growth rates and slaughter body weight were not affected by acorn incorporation in both phases ( p>0.05 ). Consequently, the carcass weights and carcass yields were similar. The F-Ac and S-Ac lambs had relatively heavier liver than F-Ba and S-Ba. The carcass composition in cutting pieces and that in tissues (muscle, fat and bone) was similar for all groups. These results suggest that acorns could replace partially conventional feedstuffs as concentrate without affecting animal performance and carcass quality.

## 1 Introduction

Animal nutrition in the southern Mediterranean area is based on natural pastures in the rangelands, where production is fluctuant among years and seasons with a particular drop of herbage availability during the dry season, resulting in inefficiencies in nutrient supply to the animals (Elloumi et al., 2011). Consequently, to supply the livestock, breeders use common concentrates and other feeds often imported and expensive. However, some local feedstuffs such as shrubs and agro-industrial and forest by-products (Obeidat et al., 2008; Mahouachi et al., 2012; Stiti et al., 2021; Yagoubi et al., 2021) could be used as a solution to overcome this shortage. The *Quercus ilex* (green oak) is one of the most common forest trees in the Mediterranean area; its product, the acorn, may serve as feedstuff in this region. It contains high starch level and tannins, which make it an alternative and cautionary feed for animals (Shimada et al., 2006). Commonly, acorns can be either grazed by the free-ranging animals or offered as a part of diets in the forest area. However, it has been used in animal feeds, mainly for pig and broiler chickens (Bouderoua and Selselet, 2003; Rey et al., 2005; Bouderoua et al., 2009; Cappai et al., 2013). Characteristics of animal products from forest could be used in order to distinguish their production systems as specific food, given the increasing interest of consumers for this category of products (Dias et al., 2008; Mekki et al., 2016). For breeders of the southern Mediterranean region, the conservation of excess of acorn for indoor use during shortage season is not a conventional practice. Furthermore the few studies on its substitution to barley in small-ruminant diet showed that up to 25 % the acorn did not influence average growth rate of lambs, while at 50 %, the nitrogen retention and the nutrient digestibility significantly decreased (Al-Jassim et al., 1998; Froutan et al., 2014). Given the availability of this forest product, the present investigation was undertaken with the purpose of its use in a rate substitution (40 %) lower than 50 % but higher than 25 % in a lamb's fattening trial. Among used animals, one-half were born and kept in the forest until weaning; they were accustomed to acorn eating; the other half came from conventional farming. The hypothesis to be verified is the following: does the acorn supply, to conventional lambs, negatively alter their growth, carcass and non-carcass traits and the diet digestibility? The meat quality of these animals was, previously, treated in Mekki et al. (2019).

## 2 Material and methods

### 2.1 Experimental feeds

The green oak acorn, the fruit of *Quercus Ilex*, was harvested in autumn from forest land of Aïn Draham in northwestern Tunisia, which is a humid region. The acorn was not dehulled, and the kernel (all the dicotyledons) was ground, within 3 d, and then dried in fresh air. The conventional concentrate is a mixture of barley, wheat bran, soya bean meal and mineral–vitamin supplement (MVS); it is considered fattening barley concentrate (F-Ba). For the experimental concentrate, 40 % of barley was replaced by acorn, so the fattening acorn concentrate (F-Ac) contains 45.6 % of barley and 30.4 % of acorn and the same proportions of wheat bran, soya bean meal and MVS as F-Ba. In addition, the concentrate cost was calculated (Table 1).

**Table 1:** Chemical composition (g kg ${}^{-1}$ dry matter, DM) and costs of experimental foods.

	Oat hay	Barley	Acorn	Concentrate – barley	Concentrate – acorn
Composition (g kg ${}^{-1}$ DM)					
Dry matter (DM)	910	960	780	900	913
Crude protein	60	80	70	130	123
NDF ${}^{1}$	659	280	288	260	258
Total phenolic content	–	–	43	–	32
Energy (kcal kg ${}^{-1}$ )	670	1230	1790	1470	1505
Constituents (g kg ${}^{-1}$ DM)					
Barley	–	–	–	760	456
Soybean	–	–	–	120	120
Wheat bran	–	–	–	90	90
Acorn	–	–	–	0	304
MVS ${}^{2}$	–	–	–	30	30
Feed cost per tonne (TND ${}^{3}$ )	425	600	200	601	479
Feed cost per tonne (USD)	157	222	74	223	178

${}^{1}$ NDF: neutral detergent fibre. ${}^{2}$ Mineral–vitamin supplement (10.0 % Ca, 3.5 % P, 8.0 % Na, 4.4 % Mg, 0.4 % S, 0.4 % Zn, 0.2 Mn, 0.2 % Fe). ${}^{3}$ TND: Tunisian dinar.

### 2.2 Experimental design, animals and diets

For this experiment, 32 male lambs from the Barbarine indigenous Tunisian fat-tail breed, weaned at approximately 4 months old and average body weight (BW) 17.2±1.5 kg, were used. They were treated for internal and external parasites and enterotoxemia at the beginning of the experiment and then were housed in individual boxes. Before and during the suckling period, 16 lambs were reared on conventional pasture; each pair mother–lamb received 400 g of barley grain as concentrate (S-Ba). The other 16 lambs were reared on forest pasture where each pair mother–lamb received 300 g of green oak acorns as concentrate (S-Ac).

During the fattening period, the lambs were assigned to four groups according to concentrate received at this phase based either on barley (F-Ba) or acorn (F-Ac) and that received on suckling phase (S-Ac and S-Ba). Hence, two groups, one from S-Ac and one from S-Ba, received F-Ac concentrate and the other two (one from S-Ac and one from S-Ba) received F-Ba concentrate. The fattening experiment lasted 90 d; during it, animals of all groups were fed oat hay ad libitum and concentrate and had free access to water. The initial amount of concentrate was 500 g, and it increased according to refused quantity. Concentrates and hay were offered in separate troughs in two equal meals at 09:00 and 14:00 local time (UTC + 1). Quantities of offered and refused feeds were recorded daily. Lambs were weighed at the beginning of the study and weekly before the morning meal. At 80 d of the growth trial, 10 animals from F-Ac and 10 from F-Ba group were transferred to individual metabolism cages for a digestibility trial, which lasted 10 d. The adaptation period to these new conditions was 5 d, followed by a 5 d period for total faecal and urinary collection. At the end of both trials (growth and digestibility), all lambs were slaughtered.

### 2.3 Digestibility measurements and laboratory analysis

During the 5 d of metabolism trial, offered hay and concentrate were weighed daily and samples conserved. Individual refusals were collected daily at 08:00, weighed, sampled, and then stored. Total daily faecal output for each animal was collected, weighed and homogenised. One sample of 100 g was dried for 24 h at 105 ${}^{\circ }$ C to measure faecal dry matter (DM), and the second of 40 g was kept at -15 ${}^{\circ }$ C. Pooled samples of faeces obtained from each animal were used for chemical analysis. Urine was collected in buckets and preserved with 50 mL of 10 % sulfuric acid. After weighing and homogenisation, aliquot fractions were pooled and stored at - 15 ${}^{\circ }$ C each day for urinary nitrogen analysis.

Samples of hay, concentrates and portions of individual pooled samples of refusals and faeces were dried (50 ${}^{\circ }$ C), ground (1 mm screen), and stored for subsequent analyses. DM was determined by drying at 105 ${}^{\circ }$ C. Total mineral content was determined by ashing at 600 ${}^{\circ }$ C for 8 h. Nitrogen (N) in hay, concentrate, faeces and urine was determined by Kjeldahl method (CP = N ×6.25 ). The methods of Van Soest et al. (1991) were applied to analyse the neutral detergent fibre (NDF) using an ANKOM220 fibre analyser (ANKOM Technology Corp., Macedon, NY, USA).

### 2.4 Measurements at slaughter, carcass cutting and dissection

Lamb BW was recorded before slaughter. After slaughter, non-carcass components, head, skin, feet, lungs, liver, heart, and all fractions of the digestive tract (reticulorumen + omasum (rumen), abomasum, and intestine) were weighed. All fractions of digestive tract were weighed full then empty after hand rinsing, in order to determine the weight of the digestive contents, whose weight was subtracted from BW to obtain empty body weight (EBW). The hot carcasses were weighed (HCW) and stored for 24 h at 4 ${}^{\circ }$ C. The cold carcass was recorded (CCW) and the subcutaneous fat thickness was measured between ribs 12 and 13 using a graduate rule. The fat tail was removed and weighed, and then the carcass was split longitudinally into two halves. The left half-carcass was cut following the procedures of Colomer-Rocher et al. (1972) into six joints (leg, ribs (thoracic region), loin, flank, neck and shoulder) which were weighed. Their proportion in the half-carcass was determined; the higher-priced joints correspond to the sum of leg, shoulder and loin. The shoulders were dissected into subcutaneous and inter-muscular fat, muscle and bone in order to estimate the tissular carcass composition.

### 2.5 Statistical analysis

For statistical analyses, the general linear model (GLM) procedure of SAS (2004) was used. For growth and carcass traits, analyses were performed based on a 2×2 factorial arrangement of suckling concentrate (S-Ba and S-Ac), fattening concentrate (F-Ba and F-Ac) and their respective interaction as fixed factors. For the digestibility parameters, only the effect of the fattening concentrate was tested.

## 3 Results

### 3.1 Nutritive value and cost of feeds

The chemical composition of the experimental feeds is given in Table 1. The crude protein (CP) was slightly higher for barley; the same observation was observed for the concentrate barley. Hence, the CP content was 130 vs. 123 g kg ${}^{-1}$ DM, respectively for concentrate barley and concentrate acorn. The NDF content and respective energy values were similar (1470 and 1505 kcal kg ${}^{-1}$ ). However, the acorn and consequently contain more total phenol (tannins).

The price of concentrate barley and concentrate acorn was USD 223 and 178, respectively (equivalent to TND 601 and 479, respectively). When barley grain was partially replaced (40 %) by acorn, the cost was reduced by 20 % (Table 1), while the nutritive value was similar for both kinds of concentrate.

### 3.2 Feed intake, nutrient digestibility and lamb growth

The daily concentrate consumption in the first week was about 450 g and the maximum 700 g. The evolution was similar with both kinds of concentrate resulting in an average intake of 630 g d ${}^{-1}$ for all groups. The hay intake was similar for all groups with a mean daily consumption of 320 g, which result in a meal with ratio forage / concentrate of 34 / 66.

The digestibility of DM, organic matter (OM), CP and NDF was similar among diets containing or not containing acorn (Table 2), averaging 57 % and 60 % for CP and NDF. The results of nitrogen balance are reported in Table 2. The N intake, urinary N, faecal N and N retention were not affected by the acorn intake and were similar for both dietary treatments ( p>0.05 ). The N balance was positive and similar for both groups with high values ( >8 g d ${}^{-1}$ ).

**Table 2:** Digestibility of diet components (%) and nitrogen (N) balance (g d ${}^{-1}$ )

Groups ${}^{1}$	F-Ba	F-Ac	SEM ${}^{2}$	p value
	N = 10	N = 10		
Digestibility (%)				
Dry matter	60.3	60.8	0.62	0.54
Organic matter	62	60.1	1.56	0.10
Crude protein	57.2	57.4	1.46	0.96
Neutral detergent fibre (NDF)	60.2	59.4	1.82	0.56
Nitrogen (N) balance				
N intake (Ni, g d ${}^{-1}$ )	15.8	15.6	0.16	0.1
N faecal (g d ${}^{-1}$ )	5.4	5.3	0.90	0.20
N urinary (g d ${}^{-1}$ )	1.7	1.9	0.19	0.12
N retained (RN, g d ${}^{-1}$ )	8.5	8.2	0.96	0.77
N efficiency (RN % Ni)	59.4	58.2	8.6	0.13

${}^{1}$ F-Ba: lambs receiving concentrate barley in fattening phase; F-Ac: lambs receiving concentrate acorn in fattening phase. ${}^{2}$ SEM: standard error of means.

The growth rate was not affected by the dietary treatment averaging 138 g d ${}^{-1}$ for all groups. The mean final body weight was 28 kg without significant differences between groups submitted to different dietary treatments (Table 3). The cost of daily weight gain of lambs was lower for F-Ac group than F-Ba group ( p<0.05 ); consequently, the feed cost of 1 kg of gain was reduced (by about 20 %) by the acorn incorporation.

### 3.3 Empty body weights, carcass and non-carcass traits

The EBW and CW were similar among groups averaging 23 and 13 kg, respectively (Table 3). Acorn incorporation at suckling and fatting phases did not affect EBW and CW; consequently, the CY averaged 45 % for all groups. However, the thickness of subcutaneous fat was significantly higher for S-Ba group than S-Ac ( p<0.05 ). The weight and proportion of organs are reported in Table 4. The intake of acorn during suckling or fatting period did not affect organ weight and their percentages ( p>0.05 ).

**Table 3:** Growth and carcass traits of lambs receiving acorn or barley diet.

Periods	Suckling ${}^{1}$		Fattening ${}^{2}$			Statistics ${}^{3}$		
	S-Ba	S-Ac	F-Ba	F-Ac	SEM	S	F	S ⋅ F
groups	N = 16	N = 16	N = 16	N = 16				
Initial body weight (BW; kg)	17.3	17.5	16.8	16.9	1.47	0.32	0.91	0.66
Average daily gain (g)	138	133	146	135	12.67	0.21	0.23	0.21
Slaughter BW (kg)	28.4	28.3	28.7	28.0	1.79	0.88	0.26	0.35
Empty BW (kg)	23.9	23.5	23.2	22.8	1.97	0.12	0.46	0.61
Carcass weight (kg)	13.0	12.8	13.2	12.7	1.00	0.27	0.03	0.45
Carcass yield (%)	45.5	45.2	45.9	45.3	3.22	0.18	0.19	0.80
Fat thickness (cm)	0.41	0.33	0.38	0.36	0.03	0.03	0.53	0.50
Cost of kg gain (USD)	1.35	1.19	1.28	1.17	0.230	0.01	0.05	0.19
Cost of kg gain (TND ${}^{4})$	3.73	3.29	3.52	3.24	0.230	0.01	0.05	0.19

${}^{1}$ S-Ba: lambs supplied barley during suckling period; S-Ac: lambs supplied acorn during suckling period; ${}^{2}$ F-Ba: lambs receiving concentrate barley in fattening phase; F-Ac: lambs receiving concentrate acorn in fattening phase. ${}^{3}$ SEM: standard error of means, S: effect of suckling diet, F: effect of fattening diet, S ⋅ F: interaction effect. ${}^{4}$ TND: Tunisian dinar.

**Table 4:** Non-carcass components: weight and proportion in empty body weight (EBW).

Periods	Suckling ${}^{1}$		Fattening ${}^{2}$			Statistics ${}^{3}$		
Groups	S-Ba	S-Ac	F-Ba	F-Ac	SEM	S	F	S ⋅ F
	N = 16	N = 16	N = 16	N = 16				
Weight (g)								
Liver	459	441	433	467	18.61	0.32	0.08	0.65
Red organs ${}^{4}$	1059	1079	1036	1106	41.96	0.65	0.12	0.31
Gut	2361	2384	2270	2290	101.4	0 .12	0.94	0.96
Skin	3203	3259	3138	3153	154.9	0.71	0.15	0.99
Head	1746	1756	1751	1753	45.6	0.23	0.11	0.50
% in EBW								
Liver	1.55	1.62	1.63	1.54	0.16	0.27	0.19	0.92
Red organs	3.81	3.84	3.87	3.69	0.43	0.59	0.25	0.40
Digestive tract	7.46	7.68	7.91	8.15	0.76	0.14	0.46	0.57
Skin	11.29	11.17	11.65	10.88	1.50	0.83	0.17	0.77
Head	5.96	6.01	6.06	5.96	0.40	0.25	0.19	0.41

${}^{1}$ S-Ba: lambs supplied barley during suckling period; S-Ac: lambs supplied acorn during suckling period; F-Ba: lambs receiving concentrate barley in fattening phase; F-Ac: lambs receiving concentrate acorn in fattening phase. ${}^{3}$ SEM: standard error of means, S: effect of suckling diet, F: effect of fattening diet, S ⋅ F: interaction effect. ${}^{4}$ Red organs: liver + lungs + trachea + heart.

### 3.4 Carcass composition

**Figure 1:**
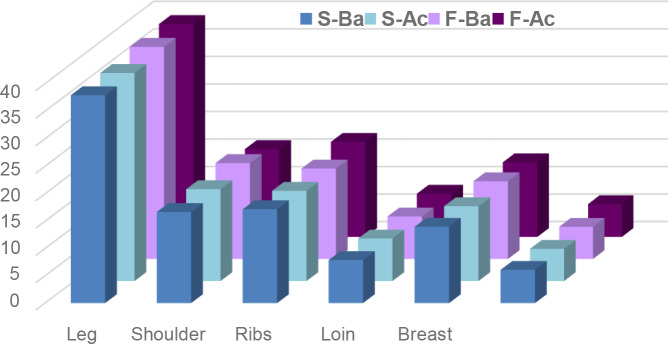
Proportion of joints in the untailed carcasses of lambs fed acorn-based concentrate (S-Ba: lambs supplied barley during suckling period; S-Ac: lambs supplied acorn during suckling period; F-Ba: lambs receiving concentrate barley in fattening phase; F-Ac: lambs receiving concentrate acorn in fattening phase).

**Figure 2:**
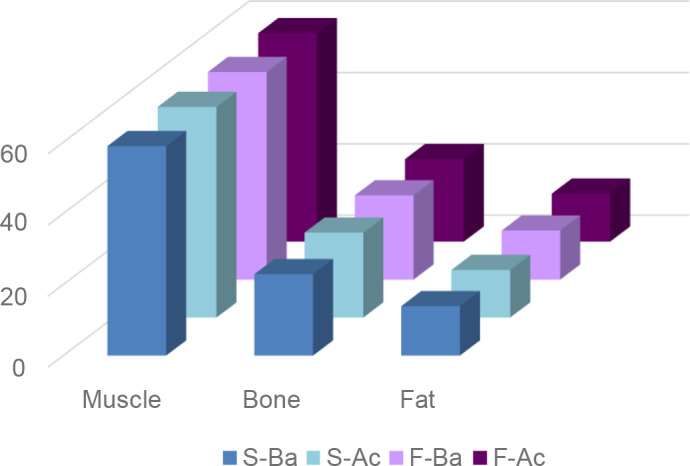
Carcass tissular proportion of lambs fed acorn-based concentrate (S-Ba: lambs supplied barley during suckling period; S-Ac: lambs supplied acorn during suckling period; F-Ba: lambs receiving concentrate barley in fattening phase; F-Ac: lambs receiving concentrate acorn in fattening phase).

The carcass regional composition was not affected by the dietary treatments. Hence, weights of leg, shoulder, ribs and lumbar regions were similar among groups. As proportions of the carcass, the main joints averaged 38 %, 17.5 % and 16.8 % of the half-carcass for the leg, shoulder and ribs, respectively (Fig. 1). In addition, the dietary treatments did not affect the carcass tissular composition as represented by the shoulder composition. The proportion of different tissues (muscle, fat and bone) in the shoulder were similar for all groups (Fig. 2).

## 4 Discussion

### 4.1 Diet intake, nutrient digestibility and nitrogen balance

The feed intake was not affected by the treatments. So, the acorn intake during suckling period was without repercussion on the concentrate intake during the fatting period. Also, the incorporation of this forest product, rich in tannin, in the concentrate did not affect its acceptability for fatting lambs. It could be concluded that the acorn is an appetising food for lambs. This result agrees with that of Al-Jassim et al. (1998), who showed that the acorn was not rejected by the animals. It also confirmed the luck of effect of acorn incorporation on total DM daily intake (Jaafari et al., 2001).

The NDF content was not affected by the concentrate kind. By contrast, Rodríguez-Estévez et al. (2010) found that the inclusion of acorns in the diet of pigs elevates the crude fibre content in the DM of diet. In the current trial the nutrient digestibility is similar, although there is a presence of tannins in the acorns, which could be attributed to diet digestibility (Al-Jassim et al., 1998). The low crude protein degradation of FA group could be also explained by the high amount of tannin in the concentrate. The low crude protein degradation of FA group could be also explained by the high amount of tannin in the concentrate (Reed, 1995; Gasmi-Boubaker et al., 2007).

For the nitrogen balance, all animals had similar N intake. In addition, no differences were observed in N loss in the faeces. Therefore, all animals were in positive N balance, F-Ac lambs have slightly higher urinary N loss when compared to the other lambs and therefore less N retention, which could be reflective of decreased digestibility. Similarly Obeidat et al. (2008) have mentioned no effects of *Prosopis Juliflora* pods in N balance of the Awassi lambs.

### 4.2 Lamb growth and costs

The cost of the concentrates was calculated based on the cost of the different ingredients and the transportation fees for acorn. Therefore, the cost of the acorn concentrate was lower than that of the barley one, resulting in a lower cost of body weight gain.

The incorporation of the acorns, albeit rich in tannins, in substitution of barley in a lamb's fattening phase did not affect the growth performance of lambs. This similarity in lamb growth resulted from the similarity in the ingested feeds. Both concentrates, with and without acorn, have similar energy and nitrogen values, so the concentrates including acorn have an equivalent nutritional value as the classical concentrate based only on barley. Furthermore, the intake of hay and concentrate was similar among the groups. Therefore, acorn intake had no repercussion on forage and concentrate intake by lambs unfamiliar with acorn (S-Ba / F-Ac). In addition, it seems to be appetising for sheep; previously Al-Jassim et al. (1998) showed that acorn was not rejected by lambs. Results of the current study on growth confirmed other results for lambs fed green oak acorn (Gadoud et al., 1992). In addition, similar results were shown for growing lambs fed with pomegranate peels (Kotsampasi et al., 2014); the authors conclude that the high concentrations of tannins may reduce intake, digestibility of protein and carbohydrates, and animal performance through their negative effect on palatability and digestion. It is important to mention that all animals finished the experiment without mortality or any sign of health problems, which confirmed the study conclusion of Al-Jassim et al. (1998).

### 4.3 Carcass characteristics

The acorn incorporation at suckling and fatting phases did not affect EBW, CW and, consequently, CY. The lack of significant effects resulted from the fact that these parameters are strongly correlated to the slaughter body weight (Sents et al., 1982; Smeti et al., 2014; Hernández-García et al., 2015; Hajji et al., 2016), which, in the present study, was unaffected by acorn incorporation.

The thickness of subcutaneous fat was significantly higher for S-Ba group than S-Ac. Hence, the acorn consumption could result in a weak fat depth and consequently lower carcass adiposity. The starch quality of concentrate including acorn resulted in the inhibition of lipogenesis, and the high lipid content of acorn diet (5.4 %) did not contribute to increasing the cover fat. This observation confirmed that of Normand et al. (2007) indicating that the lipidic contribution, even higher than 5 % in diet, did not appear to have a high incidence on the thickness of cover fat.

### 4.4 Non-carcass components

Acorn incorporation in diet did not affect the offal components of animals slaughtered at a similar BW. This result confirmed the perception that the weight of most offal components depends more on weight at slaughter rather than on the intake level or diet composition (Hajji et al., 2016; Ben Abdelmalek et al., 2019). Despite the non-significant difference, the livers of animals receiving acorn were heavier than that fed control diet. Nutrients produced by fermentation of acorn-containing diets are probably important factors in changes in liver weight (Ortigue and Doreau, 1995). The head, which content in bone is high, varied slightly with diet since it is an early maturing part (Wallace, 1948; Ben Abdelmalek et al., 2019) and less affected by the dietary effects in growing animals (Kamalzadeh et al., 1998). All groups had similar proportions of digestive tract in EBW; it was established that the digestive tract, particularly the rumen, is related to feed intake (Kabbali et al., 1992; Atti et al., 2000), which was similar for all lambs.

### 4.5 Carcass composition

As proportions of the carcass, carcass joints weights were comparable among all the treatment diets. These proportions were within the range of earlier reported results for lambs (Karim et al., 2007; Smeti et al., 2014). The proportions of carcass joint weight were similar for all dietary treatments. This result on constancy of joint proportions in the carcass is in agreement with the anatomical harmony established by Boccard and Dumont (1960) and confirmed by several authors (Sents et al., 1982; Atti et al., 2005; Obeidat and Aloquaily, 2010). Furthermore, the sum of the high-priced joints averaged 62.5 % of the half carcass for all groups. It could be concluded that replacing up to 40 % of barley in the fattening diets with alternative feeds as green oak acorn did have any impact, on cut proportions in general and high-priced joints in particular. Similarly, it was shown that carcass cuts were similar among diet treatments with substitution of sesame hulls or rosemary residue to barley as an alternative diet to lambs (Obeidat and Aloquaily, 2010; Yagoubi et al., 2021).

In the present study, the dietary treatments did not affect the relative value of muscle fat and bone. Numerically, animals of S-Ac and F-Ac had relatively less fat and more muscle. The tissue proportions depend on several factors, such as protein levels, the nature of the diet nutritional level and nutrient utilisation (Murphy et al., 1994; Mahouachi and Atti, 2005). But they mostly depend on slaughter weight, which was similar for all groups.

## 5 Conclusions

When substituted for barley in the concentrate, the acorn had an economic benefit given the lower cost of body weight gain without any health problem. It did not alter the nutrient digestibility, although this trial demonstrates the potential of acorns in diets for growing lambs without adverse effects on growth performance, indicating that concentrates containing acorn have an equivalent nutritional value to the classical concentrate based only on barley. In addition, this alternative feed did not affect the carcass and non-carcass characteristics. However, its effect on carcass adiposity needs more research; furthermore and considering its richness on anti-oxidants, its impact on lipid oxidation and fatty acid profile is interesting.

## Data Availability

The original data of the paper are available upon request to the corresponding author.

## Code availability

The software code of the paper is available upon request to the corresponding author.
